# A Proposed New Nomenclature for the Immunoglobulin Genes of *Mus musculus*

**DOI:** 10.3389/fimmu.2019.02961

**Published:** 2019-12-18

**Authors:** Christian E. Busse, Katherine J. L. Jackson, Corey T. Watson, Andrew M. Collins

**Affiliations:** ^1^Division of B Cell Immunology, German Cancer Research Center, Heidelberg, Germany; ^2^Immunology Division, The Garvan Institute of Medical Research, Darlinghurst, NSW, Australia; ^3^Department of Biochemistry and Molecular Genetics, University of Louisville, Louisville, KY, United States; ^4^School of Biotechnology and Biomolecular Sciences, University of New South Wales, Sydney, NSW, Australia

**Keywords:** immunoglobulin, nomenclature, V genes, B cell, IGH, IGK, IGL

## Abstract

Mammalian immunoglobulin (IG) genes are found in complex loci that contain hundreds of highly similar pseudogenes, functional genes and repetitive elements, which has made their investigation particularly challenging. High-throughput sequencing has provided new avenues for the investigation of these loci, and has recently been applied to study the IG genes of important inbred mouse strains, revealing unexpected differences between their IG loci. This demonstrated that the structural differences are of such magnitude that they call into question the merits of the current mouse IG gene nomenclatures. Three nomenclatures for the mouse IG heavy chain locus (*Igh*) are presently in use, and they are all positional nomenclatures using the C57BL/6 genome reference sequence as their template. The continued use of these nomenclatures requires that genes of other inbred strains be confidently identified as allelic variants of C57BL/6 genes, but this is clearly impossible. The unusual breeding histories of inbred mouse strains mean that, regardless of the genetics of wild mice, no single ancestral origin for the IG loci exists for laboratory mice. Here we present a general discussion of the challenges this presents for any IG nomenclature. Furthermore, we describe principles that could be followed in the formulation of a solution to these challenges. Finally, we propose a non-positional nomenclature that accords with the guidelines of the International Mouse Nomenclature Committee, and outline strategies that can be adopted to meet the nomenclature challenges if three systems are to give way to a new one.

## Introduction

The generation of antibody diversity relies in part on the use of genes from extensive gene families residing in the immunoglobulin (IG) loci of the mammalian genome. Remarkably, a comprehensive understanding of the organization of these gene sets emerged long before a detailed knowledge of antibody gene sequences was available (1, 2), and much of the research that led to this understanding was performed in mice. The IG gene loci – being polymorphic and polygenic—are especially complex, which has created challenges for the development of a gene nomenclature that is both logical and sustainable. This manuscript presents a new proposal to meet this challenge.

Mouse antibody polypeptide chains, and the genes that encode them, were first identified using mineral-oil induced plasmacytoma cell lines derived from BALB/c mice (3). It was soon realized that functional mouse IG heavy and kappa light chain variable (*Ighv, Igkv*) genes exist as multigene families (4, 5). *Ighv* genes, e.g., belong to 15 gene families (5, 6), and the first names given to the *Igkv* and *Ighv* gene families came from the names of the cell lines that were used in their identification. For example, the anti-dextran antibody-producing cell line J558 was used to generate a DNA probe by which a family of *Ighv* genes was identified by Southern blot analysis (7). This family was subsequently termed “J558,” and today is generally known as the *Ighv1* family.

*Ighv* gene probes were also used in Southern blot analysis to explore the loci of different inbred mouse strains, with eight different haplotypes being identified (7). This expanded a system of classification that began with serologically-defined allotypic variation in the immunoglobulin constant regions (8). In this system, the BALB/c and C57BL/6 *Igh* haplotypes were designated *Igh*^*a*^ and *Igh*^*b*^, respectively.

Although a later investigation of 72 inbred mouse strains found that 13 strains carried variant haplotypes involving substantial structural variation (9), broad similarities were believed to exist between the *Igh* loci of strains carrying shared haplotypes (6, 10). After the sequencing and annotation of the *Igk* locus (11, 12) and the *Igh* locus (13, 14) of the C57BL/6 strain, the earlier Southern blot studies provided justification for comparisons of sequences from other strains with those of the C57BL/6 reference genome, and for the identification of sequences as allelic variants of their most similar sequences in the reference genome. The sequencing of the loci also led to the development of new nomenclatures for both the heavy (14–16) and the light chain (17). Discussion here will focus on the nomenclature of the variable genes of the heavy chain, *Ighv*.

**Figure 1:**
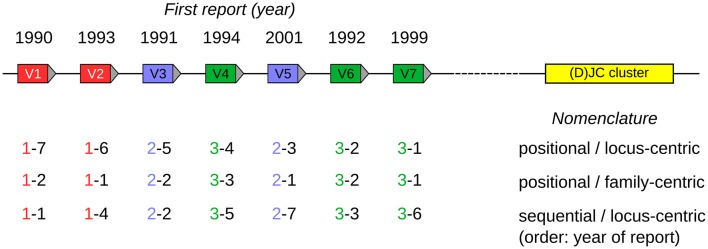
Visualized scheme of three nomenclature strategies, using a hypothetical locus encompassing seven V genes (labeled V1–V7) belonging to three V gene families (indicated as red, blue, green). The year of the first report is indicated above the genes. The (D)JC region is shown as a yellow box and provides orientation for the positional strategies. The designations beneath the individual V genes follow the <family>–<member> format discussed in the text. To increase the readability, the <stem> component has been omitted from the designations, as it would be identical for all designations, since only a single locus is shown here. For better clarity, gene family designations are also indicated by text color.

A positional nomenclature was developed by the International ImMunoGeneTics Information System (IMGT) group (18), based upon the mouse genome reference sequence (C57BL/6), in which each gene was assigned a name reflecting its gene family, and the sequential position of the gene within the locus from proximal to distal positions, relative to other genes of that family. The most proximal gene of a family was given the position number “1,” counting up to the most distally located gene in that family, which was given the number equalling the total number of genes in that family (a scheme referred to as *family-centric* below, also see Figure 1). This is different to the IMGT nomenclature for human *IGHV* genes, in which the position number refers to the position of the gene within the entire set of *IGHV* genes, with the most proximal gene being numbered “1,” and the most distal gene being numbered “81” (referred to as *locus-centric* scheme below). In the IMGT nomenclature, the locus name is included in the gene name (e.g., IGHV1-18), and the old *Ighv* family names are replaced with a numbering system proposed by Honjo and Matsuda (19).

A positional nomenclature was also developed by Johnston and colleagues, based upon their alternative genome assembly of the C57BL/6 *Igh* locus (14). The Johnston nomenclature utilizes the earlier gene family names (7183, J558, 36-60, etc.), a number representing the position of the gene within the gene family, and a second number representing the position of the gene amongst all genes of the locus (e.g., J558.31.121, 7183.7.10). In this nomenclature, pseudogenes are indicated by an additional “pg” tag (e.g., 36-60.7pg.72). A study of the *Ighv* locus of the 129S1 strain led to the development of a variant of the Johnston nomenclature by Retter et al. (16). While still following the basic rules set by Johnston et al., Retter et al. constructed the names using a locus descriptor (“VH”), the earlier *Ighv* gene family name, a letter referring to the *Igh* haplotype of the inbred strain, a number representing the position of the gene within the gene family, followed by the “psi” tag for pseudogenes, and a second number denoting the position of the gene within the locus (e.g., VH7183.a3psi.5). Both the Johnston and the Retter reference data sets can be readily accessed for analysis e.g., via IgBLAST (20). Finally, while Retter and colleagues also developed a further designation system for their VBASE2 sequence repository (21), it should be noted that we consider these to be primarily sequence identifiers, rather than a genetic nomenclature in the strict sense.

All three mouse nomenclatures are currently in use, and all are challenged by recent findings that show that there are substantial differences, including structural differences, between the *Igh* loci of different classical inbred mouse strains (22). It has been proposed that the differences between the genes of the BALB/c and C57BL/6 strains could have resulted from their loci having originated in different subspecies of the house mouse (22). However, investigations of *Ighv* genes in wild-derived strains now suggest this is not the case (23). Instead, the *Ighv* loci of classical inbred strains appear to be mosaics, made up of many relatively short haplotype blocks that may have their origins in disparate subspecies of the house mouse, and even in other *Mus* species.

The discovery of variation in BALB/c and wild-derived strains was made using inferential techniques. These techniques are widely used in human and other species to identify allelic variants (24–26), but it is already clear that it will not be possible to unequivocally associate most inferred variants in the mouse to any particular gene in the C57BL/6 genome reference sequence (22, 23). For example, amongst the set of inferred BALB/c *Ighv* sequences, there are instances in which three or more sequences are most closely aligned with a single C57BL/6 *Ighv* gene. In such circumstances, it is impossible to discern whether these BALB/c sequences represent allelic variants of the C57BL/6 genes or distinct gene loci.

It therefore has become increasingly clear that, given the extent of IG diversity likely to be encountered among commonly used inbred mouse strains, our ability to effectively characterize and catalog mouse IG genes and alleles will be constrained by the current nomenclature systems. There is therefore a clear requirement for a new, more flexible nomenclature that will better meet the needs of the community. Here, in light of the challenges we face with the curation of mouse IG sequencing data, we discuss the key aspects that should be considered in the establishment of any nomenclature system. We use this discussion to motivate the proposal of a new non-positional mouse IG nomenclature.

## Consideration for a New Mouse IG Nomenclature

### Challenges and Constraints

**Figure 2:**
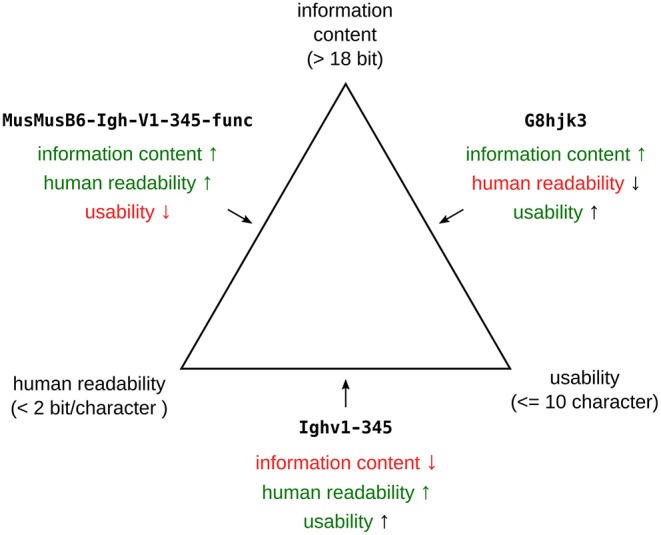
Ternary plot depicting the constraints for gene symbols. The individual properties and their boundaries are located on the three corners. The minimal information content is based on the requirement to be able to encode at least 4 loci, 3 different types of gene segments (V, D, J), 32 gene families and 1024 members. The 10 character limit (usability) is based on current IMNC guidelines. The limits for human readability are a compromise between standard English language entropy (≈ 1 bit per character) and pure numerical representation (3.3 bit per character). Examples that optimize two properties (shown in green) at the expense of the third one (red) are shown on the median of the respective edges.

Before considering the specifics of *Igh* nomenclature, it is worthwhile to consider what gene symbols (27) and their use must, should and should not try to achieve. Note that gene names (27) (e.g., Immunoglobulin Heavy Variable 1–2) will not be discussed separately here, as they are rarely used in scientific communication, and gene symbols (e.g., IGHV1-2) are synonymous with them. Gene symbols in general aim to provide designations to hereditary units, which in virtually all cases refer to specific physical regions in the genome. This nowadays often translates into linear base-pair sequences. Gene symbols serve as handles for this information, and have to balance three interdependent properties (Figure 2): a symbol should be unique (i.e., refer to a single specific gene), human-decodable and short enough for everyday use. The criterion of human-decodability requires a formalized system, e.g., that all IG symbols start with “Ig.” This creates redundancy and thus reduces the potential information content. In combination with the limited length for a symbol—the International Committee on Standardized Genetic Nomenclature for Mice (IMNC) [https://perma.cc/6F9S-6H4U] recommends a maximum of ten characters (27, 28)—this means that the overall information content of a symbol is limited. From this it follows that a gene symbol should encode only the minimal information required for the unambiguous identification of each particular gene. We will refer to this conclusion as the *lean designator principle* below.

Based on these theoretical limitations, we now need to consider what information a gene symbol should not attempt to encode. Firstly, a gene symbol is not required to be a synonym for a specific physical location on the genome (e.g., “Chromosome 12; BPs 114,048,536-114,048,547”). Indeed, before the current era, in which the complete sequencing of genomes is now commonplace, having a fine-grained physical mapping for a gene in an organism was the exception rather than the rule. Secondly, a gene symbol is not expected to be used without some biological context. This implies, on the one hand, that it should not encode information that can be stored and accessed elsewhere using the gene symbol as identifier (e.g., from a reference database). On the other hand, it means that a gene symbol is not a globally unique identifier, but only a unique identifier within a single organism, as it is safe to assume that the biological context provides knowledge of the species. Thirdly, harmonizing gene symbols between organisms has been a long-standing but ultimately futile endeavor. It is critical to recognize that any attempt at harmonization is at variance with the *lean designator principle*, as it tries to encode non-essential information about communality within a gene symbol.

With this basic theoretical understanding regarding the general design of gene symbols, we now need to understand how these symbols are best assigned to real-life data. While the following considerations can—in theory—be applied to all genetic loci, they are clearly most relevant to loci harboring large ensembles of genes from one or multiple related gene families (e.g., immunoglobulins). We will refer hereafter to these loci as “polymorphic, polygenic and repetitive loci” (PPRL). As discussed in the Introduction, various individuals of a species can exhibit substantial diversity in the form of single nucleotide polymorphisms (SNPs) as well as larger structural variants (SVs; e.g., deletions and duplications). Therefore, the sequencing of a PPRL in a new individual will often result in the observation of novel sequence variants for a number of genes. The frequently employed naming paradigm for such new genes is to *lump*, i.e., to assign the gene symbol of the closest related known gene to the novel sequence. However, this operation implies that it is most likely that the two sequences are allelic variants of the same gene, which is a claim that should require evidence in its support or otherwise be rejected. Importantly, with the increased structural variation observed in PPRL, the alternative scenario of a paralogous relationship existing between two observed sequences not only becomes more likely, it also becomes more challenging to detect due to the high redundancy of the surrounding sequences. In these cases, the opposite naming paradigm of *splitting*, i.e., assigning new gene symbols to a novel sequence, unless the allelic relationship of two sequences is proven beyond doubt, should be the preferred mode of action. While both naming paradigms can exhibit false-positive and false-negative errors, the key consideration should always be to protect the integrity of the scientific record in the most reliable manner. The consequence of these different errors is illustrated by the following complementary scenarios:

- False-negative gene assignment: a novel sequence is observed, which has a high but not perfect homology to a known gene. Under the *lump* paradigm, the sequence is assigned the gene symbol of the closest related known gene and considered to be an allelic variant of it. Five years later, it is recognized that the two “alleles” instead represent distinct genes. Therefore the more recently characterized sequence is given a new gene designation and the initially assigned allele symbol is retired. All past scholarly communication that does not clearly provide an allele designation will require reconsideration as the report could be referring to either gene. Continued use of the initial faulty nomenclature will continue to create confusion.
- False-positive gene assignment: As above, a novel sequence is observed, which has a high but not perfect homology to a known gene. Under the *split* paradigm, the sequence is assigned a new gene name. Five years later, it is recognized that the two “genes” actually represent alleles of the same gene. Therefore the initial designation is now retired and the newly assigned allele symbol is linked to the older designation. All scholarly communication using the erroneously assigned gene symbol can be easily understood. Continued use of the erroneously assigned designation is bad practice but not harmful.

These examples show that the potential loss of information regarding “inheritance by descent” in the *split* paradigm can be dealt with more easily than the lack of accuracy imposed by the *lump* paradigm.

The complexity of PPRLs might also require a revision of the current allele designation strategy: The IMNC currently assigns lower-case letters to mouse alleles of all loci, which are based on the reference strain in which a given sequence is observed. Importantly, this system mixes allele information with haplotype information. While this might be appropriate for stable parts of the genome, recent studies (23) suggest that novel haplotypes of the *Igh* locus will likely be identified with the analysis of each new inbred strain. This creates a situation in which multiple distinct haplotypes can share the same sequence. This is an example of the inappropriate use of gene symbols whereby too much information is being encoded in too little space, based on the assumption of relatively high stability and homogeneity between strains. Haplotypes are better stored in reference databases and/or the metadata for an allele, rather than in the allele symbol. We believe that this should be implemented in any new IG allele nomenclature.

Finally, it should be noted that gene symbols should follow the general nomenclature guidelines for a given species. This not only reduces potential ambiguity in scholarly communication and facilitates simplified distinctions between species (e.g., human and mouse), but it also allows for automated formatting. Of note, the nomenclatures of Johnston et al., Retter et al. and IMGT all fail to comply with IMNC guidelines. The nomenclatures of Johnston et al. and Retter et al. use punctuation, while the IMGT nomenclature uses gene symbols in all-caps with a numeric representation of alleles. All these features are at variance with IMNC guidelines.

In summary, we hope that we have established five central aspects for the curation of sequences in PPRL. Firstly, gene symbols need to be human-decodable, hence overall information needs to be minimized. Secondly, the best way for gene symbols to be human-decodable is to consider them to be designations for sequences, and nothing else. Thirdly, gene assignments should in general follow the *split* paradigm, as it is more robust to changes over time. Fourthly, the IMNC-recommended mouse allele nomenclature needs revision. Finally, gene symbols need to follow the established nomenclature rules for a given species.

### Potential Numbering Strategies

The IMNC-recommended way to assign symbols to individual genes of a gene family follows a <stem><family><member> format (27), in which the <stem> field denotes the super-group of genes, the <family> field indicates the gene family and <member> the individual gene. The <stem> gene super-group usually closely follows the common concept of a “gene locus,” e.g., *Ighv*, the special case of off-loci genes will be discussed separately below. However, there is no common standard stating whether <family> and <member> should be represented by letters or Arabic numerals: Protocadherins (*Pcdh*) use a “Letter Number” format, olfactory receptors (*Olfr*) use “Number Letter Number” in humans, but only numbers in mice. The Human Genome Nomenclature Committee (HGNC)-approved IMGT naming scheme for human IG and TR uses a “Number – Number” format, in which the fields are separated by a hyphen (the usage of which is explicitly allowed by HGNC for these loci). As there is no general problem with this format, we believe this component of the nomenclature should be retained.

The assignment of individual genes to families is usually based on arbitrary thresholds of sequence homology. However, it should be noted that this assignment procedure constitutes a non-trivial partitioning problem, especially when facing an increasing number of elements. As a detailed discussion of this problem is beyond the scope of this manuscript, we will assume that these assignments can be performed in a deterministic and stable fashion. An assignment is considered deterministic if each element will always be assigned to the same family, and stable if the addition of an *n* + 1^*th*^ element does not alter the assignments of any of the other *n* elements.

There are multiple ways in which the number in the <member> field can be assigned and most of the existing strategies reflect differing responses to two questions:

1. Should the <member> field indicate the position of the gene in its locus, based on a reference genome assembly (“positional”), or just be assigned in an incremental way (“sequential”), e.g., according to the order of discovery?
2. Does the <member> field partition the namespace of all genes of the locus (*locus-centric*) or just the namespace of the members in a given family (*family-centric*)?

In the past—as discussed in the introduction—a multitude of approaches have been used for PPRL in general and for the IG loci in particular. These are depicted in a schematic way in Figure 1.

It is critical to recognize that positional schemes are problematic in general and especially when used for nomenclature of PPRL. Firstly, they violate the *lean designator principle* as by definition they encode positional information that is not strictly necessary for the gene symbol. Secondly, as they struggle to deal with duplications and other additive SVs, they are not well suited for application of the *split* paradigm, which we have established is appropriate for any PPRL nomenclature. Thirdly, not only do they encourage the use of the *lump* paradigm, but they also fail catastrophically once a downstream *split* is required. This is due to the fact that a *split* within an—initially—positional scheme requires an extension of the format to perform the required subpartitioning, which then by itself can lead the positional numbering *ad absurdum* (e.g., if *Ighv1-23a* and *Ighv1-23b* are not located next to each other). Finally, positional schemes usually assume that all genes are located in a single continuous locus, which—as discussed below—might not be the case. Because of these problems, we believe that only a sequential scheme can provide the flexibility required by PPRLs.

In regard to the question of which space the <member> field should actually partition, we favor a *locus-centric* scheme as this means that the <member> field becomes a unique identifier for a given gene, independent of the gene family assignment. This not only provides for more error-tolerant designations, as no two families share a gene with the same <member> field, but it also allows for more flexibility should a reassignment of families become necessary.

Having decided on a sequential and *locus-centric* scheme, we must now consider the actual assignment procedure that would be performed once a novel sequence is observed. As we have argued before, we should assume incomplete knowledge of the locus structure and therefore in general follow a *split* rather than a *lump* paradigm. Assuming that a novel sequence can always be grouped into a family, a new member number should therefore be assigned by default. However, this does not mean that all pre-existing information needs to be rejected. Taken to the extreme, such a rejection would mean that sequences from a well-known line of an inbred mouse strain that had been kept for numerous generations at a particular facility, would all need to be assigned new gene designations, as there may have been genetic divergence of the colony since its founding. We therefore consider it prudent to introduce a principle of parsimony, which implies that above a certain threshold, “Identity by descent” of two sequences will be considered to be likely. To maintain the stringency of this approach, we propose setting the threshold at 100% identity of the coding sequence. This allows on the one hand to collapse the majority of sequences observed when re-sequencing lines of existing strains but on the other hand follows the *split* paradigm as closely as possible. While thresholds slightly below 100% might seem attractive, as they could accommodate potential sequencing errors, we reject such thresholds as being arbitrary and situations are known to exist where two genes reside at distinct genomic locations but differ by just a single nucleotide. Furthermore, we consider both sequencing and inference technologies to be advanced enough by now that appropriate error correction should be in place.

In summary, we here propose a sequential and *locus-centric* nomenclature based on a parsimonious *split* paradigm.

###  Handling of Existing Designations

The introduction of a revised nomenclature naturally raises the question of how to handle legacy designations. In general, as the current IMGT designations of C57BL/6 genes do not contain any obvious errors, these names should remain in place. However, all other alleles that are not present in GRCm38 should be subject to renaming, based on the scheme described here. The strict use of IMNC formatting will avoid potential confusion by clearly distinguishing legacy IMGT names from revised names. In addition, use of an initial value for the <member> field of 200 or above would avoid collisions in cases where the IMNC formatting is not used.

The proposed consecutive assignment of gene numbers might for some readers be reminiscent of the “S”-nomenclature used by IMGT for many unmapped sequences. This could lead to the perception that the “S”-nomenclature might present a more conservative way of addressing the existing nomenclature problems than the much more drastic revised nomenclature proposed here. However, it should be pointed out that IMGT considers the “S”-nomenclature to be a temporary designation that precedes the assignment of a positional-based gene symbol. In contrast to this, the nomenclature proposed here rejects the presumption that we will be able to map relevant genes with sufficient certainty.

The nomenclature for so-called “orphaned” V genes also needs to be addressed. These are genes residing at substantial distance from the main gene loci, often on other chromosomes. There are two general strategies that might be used to handle these genes, based on the concept of what the <stem> field refers to. On the one hand, the <stem> field could designate a single and continuous physical location in the genome. In this case, e.g., the “Igh” prefix would be considered a shorthand for “Chromosome 12; BPs 113,225,000-116,024,999,” and all genes outside of this region would bear another designation. Based on strategies used for other gene families, these genes could for example be prefixed as “Ighvl” (“Immunoglobulin heavy variable-like”). On the other hand, <stem> could be considered a designation that a gene is part of a super-group of gene families (based on homology), with information about the physical location being stored elsewhere. In this case, “orphaned” genes would use the usual <stem> (e.g., “Ighv”), a <family> number based on the general homology thresholds as discussed above and unique < member> number. It should be noted that IMNC defines a “locus” as a mappable “point in the genome” (27) and both concepts of the <stem> field are compatible with this definition. The main differences between the two concepts are based on the interpretation of a locus as a continuous region vs. the grouping of genes based on homology independent of their location in the genome. As the currently available data does not show support for any claimed utilization of “orphaned” genes in V(D)J rearrangements, we think that *lean designator principle* is eclipsed by the aim of a stricter definition of the locus. Therefore we would argue for the reassignment of the off-loci genes as “-likes.”

## The Way Forward

The challenges to existing nomenclatures that stimulated this manuscript were studies that identified new mouse IG genes by inference from rearranged V(D)J sequences, rather than new genes that were identified by genomic sequencing. As we expect that the inference process will likely dominate mouse IG gene studies for some time, we believe that the development of a new nomenclature should go hand in hand with the development of a system for the validation of inferences by the research community. Procedures have recently been established for the validation of genes of the human *IGH* locus, through the establishment of the Inferred Allele Review Committee [IARC; (29)]. We would like to propose the creation of a Mouse Immunoglobulin Gene and Allele Review Committee, in cooperation with IMGT, IMNC and the AIRR Community, and under the auspices of the International Union of Immunological Societies (IUIS) Nomenclature Committee.

In conclusion, we hope that this proposal will stimulate discussion among and action by the stakeholders involved in the mouse IG nomenclature, to resolve these critical issues. Long live *Igh*!

## Author Contributions

CB and AC drafted the manuscript. All authors contributed to the editing of the manuscript and contributed to the development of the procedures described.

### Conflict of Interest

The authors declare that the research was conducted in the absence of any commercial or financial relationships that could be construed as a potential conflict of interest.
